# How to put a hex on HOX

**DOI:** 10.1002/hem3.83

**Published:** 2024-06-03

**Authors:** Robert K. Slany

**Affiliations:** ^1^ Department of Genetics Friedrich‐Alexander‐University Erlangen‐Nürnberg Erlangen Germany

HOX‐homeobox transcription factors are best known for their prominent role in embryogenesis where they control body segment identity. This regulatory principle has been “recycled” in adult tissues. HOX proteins frequently regulate the differentiation of tissue stem cells and aberrant HOX function can induce derailed maturation and tumorigenesis. A paradigm for this principle is hematopoiesis where HOXA9, a member of the so‐called abdominal HOX proteins, has acquired a notorious reputation for its frequent involvement in leukemogenesis. A number of recurrent genomic aberrations in acute leukemia are associated with elevated HOXA9 expression. Examples are KMT2A (MLL) and NUP98 fusion proteins as well as the very common NPM mutations, which all induce HOXA9 overproduction. Besides, a sizable portion of acute myeloid leukemia with normal karyotype shows abnormally high levels of HOXA9. Overall, HOXA9 dysregulation can be observed in more than 50% of all cases of myeloid leukemia, and generally, this is associated with a negative prognosis (Figure 1).^1^

**Figure 1:**
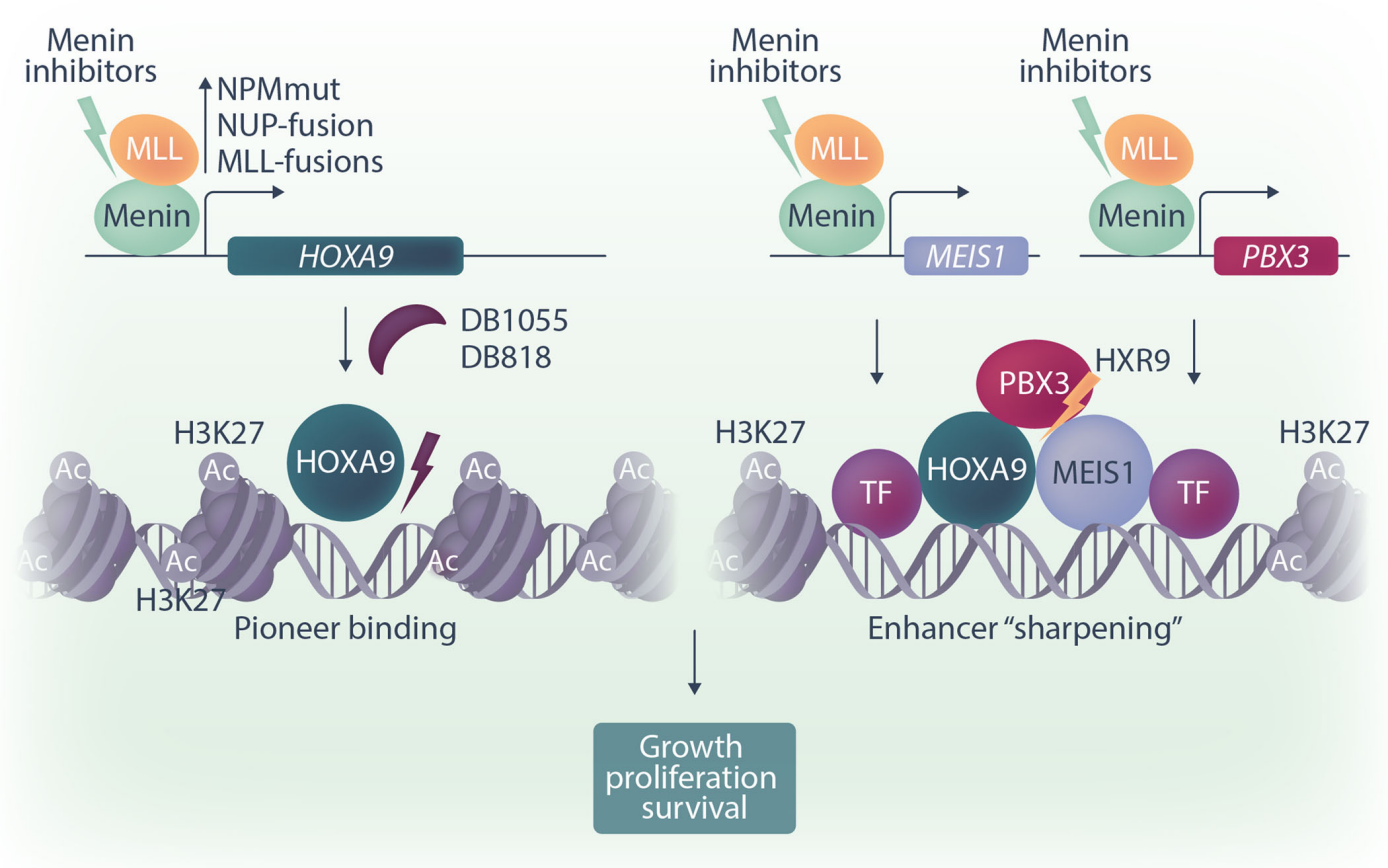
HOXA9 function and principles of HOXA9 targeting. HOXA9 is a pioneering transcription factor that can prepare chromatin for the establishment of developmental enhancers. On a subset of initial binding sites, higher‐order complexes of HOXA9 with MEIS1 and PBX3 as well as other hematopoietic transcription factors assemble and demarcate high‐activity enhancers (enhancer sharpening) necessary for self‐renewal and proliferation of precursor cells. Strategies to interfere with this process include the prevention of HOX/cofactor expression by menin inhibitors, peptides that block multimer formation (HXR9), and, as a new strategy, small molecules that interfere with HOXA9 binding to DNA (DB818/1055).

At a molecular level, HOXA9 acts as a so‐called pioneer transcription factor that binds to a variety of AT‐rich binding sites that mark prototypical enhancers and promoters important for hematopoietic precursor cells.^2^ On a subset of these sequences, HOXA9 assembles a trimeric complex with two other homeodomain transcription factors MEIS1 and PBX3, which contribute further DNA binding specificity. This stabilizes the trimer and in conjunction with other transcription factors leads to the establishment of specific enhancer/promoter sequences in a process that has been called enhancer sharpening.^3^ In consequence, genes necessary for the growth, proliferation, and survival of hematopoietic precursor cells are strongly activated. Prominent examples of HOXA9/MEIS1/PBX3 targets are *MYB*, *MYC*, *CDK6*, *BCL2*, and ribosomal genes to name just a few.^4^, ^5^ This explains why the constitutive expression of HOXA9 is such a strong cancer driver. In normal cells, the production of HOXA9 and its binding partners is extinguished during differentiation.

These properties make HOXA9 an attractive target for pharmacological intervention. Unfortunately, transcription factors are notoriously hard to target with pharmaceutically applicable substances.^6^ The most advanced attempts to derail the HOX network relies on an indirect approach by blocking the function of menin.^7^ Menin is the product of the gene *multiple endocrine neoplasia* and it physically binds to the histone methyltransferase KMT2A and its fusion derivatives. This association is necessary for the proper localization on chromatin. Menin inhibitors disrupt the menin KMT2A interaction and show early clinical promise in KMT2A‐rearranged and NPM‐mutated leukemia. For unknown reasons, however, not all KMT2A target genes are equally dependent on menin. Menin inhibition is less efficient in suppressing *HOX* expression and seems to be more effective for other KMT2A targets. In addition, cells require rapid treatment resistance, probably mandating combination therapies. A more direct approach to HOX blockade was developed more than a decade ago with a cell‐permeable peptide called HXR9. HXR9 was modeled after the HOX/PBX interaction surface and this peptide sterically competes for HOX/PBX complex formation. Administration of HXR9 showed promise in tissue culture experiments, yet clinical trials with HXR9 have not been published so far.

A new and potentially very promising approach to target HOXA9 is described by David‐Cordonnier and colleagues in this issue of *Hemasphere*.^8^ This work is based on a previous screen performed by the authors where they searched a library of heterocyclic diamidines that are known to have minor grove DNA binding capacity. They identified two compounds (DB818, DB1055) with an affinity for the common core HOX binding sequence 5′‐ATTTA‐3′. The current manuscript highlights the promise of these substances as potential HOX DNA‐binding competitors. Administration of low micromolar concentrations of DB818/1055 affected proliferation, self‐renewal capacity, and viability of a panel of leukemia cell lines with a clear correlation to endogenous *HOXA9* expression. Concomitant with the role of HOXA9 as a major determinant of an immature cellular state, treated cells also initiated differentiation. Most remarkably, and despite the undisputed importance of HOXA9 also in normal hematopoietic development, the authors could not detect any detrimental effect of DB818/1055 treatment on normal CD34+ hematopoietic stem cells (HSCs) in vitro.^8^

These results are complemented by an exhaustive set of side‐by‐side experiments comparing the lentiviral knockdown of HOXA9 with the administration of DB818/1055. Unfortunately, technical issues precluded the demonstration of a physical exit of HOXA9 from chromatin after the addition of DB818/1055 by chromatin immunoprecipitation. Yet, gene expression changes and chromatin accessibility studies suggested that DB818/1055 selectively affected the expression of previously identified HOXA9 target genes. In addition, competition of DB818/1055 and HOXA9 for DNA could be convincingly demonstrated in biochemical assays.^8^

In a further series of experiments, DB818/1055 was compared to the knockdown of HOXA9 in a THP‐1 cell line transplantation model where both approaches affected a moderate but significant prolongation of disease‐free survival. Finally, the in vivo efficacy of DB818/1055 was also visible in xenotransplants with injected patient blast cells. In these experiments, DB1055 could reduce the outgrowth of human leukemia comparable to a cytosine arabinoside test treatment.^8^

In summary, this work shows impressively that direct targeting of the DNA binding activity of a HOX transcription factor is feasible. Like always, caveats remain. Apart from the fact that it may be difficult to achieve micromolar concentrations in a patient setting, a lack of specificity for HOXA9 may be a concern. The core 5′‐ATTTA‐3′ sequence is a common affinity determinant for many different HOX homeobox genes. Removing pioneering HOX factors from DNA will affect a wide range of HOX‐containing complexes beyond HOXA9. Remarkably, the authors did not observe any effect on the development of HSCs in vitro, or on animal physiology in transplantation experiments. The relatively short treatment periods may be the key here, as one would expect widespread and systemic effects after HOX blockade, particularly on hematopoiesis. In real life, this may be less problematic as experience shows that administration of pharmacons with similarly broad effects, like histone (de)acetylase inhibitors, menin inhibitors, and others can have a therapeutic window. Thus, direct targeting of HOXA9 and other HOX proteins may be a valuable new weapon in the arsenal to treat acute leukemia.

## AUTHOR CONTRIBUTIONS

Robert K. Slany wrote the manuscript.

## CONFLICT OF INTEREST STATEMENT

The author declares no conflict of interest.

## FUNDING

Work in the laboratory of the author is supported by Deutsche Forschungsgemeinschaft (SL27/9‐2) and Deutsche Krebshilfe (70115901).
